# Predicting Insulin Resistance in Taiwanese Men Using Machine Learning: An Integrated Analysis of Biochemical Markers and Volatile Organic Compounds

**DOI:** 10.3390/biomedicines14081751

**Published:** 2026-08-03

**Authors:** Yung-Sheng Cheng, Dee Pei, Ta-Wei Chu, Shih-Ming Kuo, Yao-Jen Liang

**Affiliations:** 1 Division of Pediatric Surgery, Department of Surgery, Tri-Service General Hospital, National Defense Medical University, Taipei 114202, Taiwan; happyglenndmc.yungsheng@gmail.com (Y.-S.C.); kuoshihming@yahoo.com.tw (S.-M.K.); 2 Graduate Institute of Applied Science and Engineering and Department & Institute of Life Science, Fu Jen Catholic University, New Taipei City 242062, Taiwan; 3 Division of Endocrinology and Metabolism, Department of Internal Medicine, School of Medicine, College of Medicine, Fu Jen Catholic University Hospital, Fu Jen Catholic University, New Taipei City 243089, Taiwan; peidee@gmail.com; 4 Department of Obstetrics and Gynecology, Tri-Service General Hospital, National Defense Medical University, Taipei 114202, Taiwan; taweichu@gmail.com; 5 MJ Health Research Foundation, Taipei 114066, Taiwan

**Keywords:** insulin resistance, machine learning, volatile organic compounds, XGBoost, HOMA-IR, biomarkers

## Abstract

**Background:** Type 2 diabetes (T2D) and insulin resistance (IR) are major global health challenges. Volatile organic compounds (VOCs) in exhaled breath offer a non-invasive window into metabolic dysregulation. This study aimed to predict HOMA-IR using machine learning (ML) by integrating biochemical markers and VOC profiles in a male cohort. **Methods:** This cross-sectional study included 1258 male participants from the Taiwan MJ cohort. Four ML algorithms (Elastic Net, MARS, Random Forest, and XGBoost) were trained to predict HOMA-IR. Model performance was evaluated using R^2^, RMSE, and MAE. SHAP analysis was used to interpret feature contributions. **Results:** Ensemble tree-based approaches (Random Forest and XGBoost) demonstrated better predictive performance than Elastic Net and MARS. Random Forest achieved the highest predictive performance on the test set (R^2^ = 0.323). SHAP analysis identified BMI (mean |SHAP| = 1.326) as the strongest predictor, followed by TG (1.005) and HDL-C (0.445). Notably, specific breath VOCs, including methanol and formic acid, ranked among the top 20 predictors, capturing distinct aspects of metabolic dysregulation orthogonal to standard blood tests. **Conclusions:** Integrating VOC profiles with clinical markers provides acceptable predictive performance for IR in men. While traditional metabolic markers dominate the prediction, specific VOCs capture distinct metabolic information, highlighting the potential of breath analysis as a complementary early screening tool.

## 1. Introduction

Type 2 diabetes (T2D) is a pressing global health challenge, with prevalence projected to reach 1.3 billion by 2050 [1]. In Taiwan, diabetes prevalence shows a concerning trend, particularly among middle-aged and older adults, where the vulnerability to metabolic decline is highest: prevalence among males aged 65–74 years reaches 34%, and nearly 43% for those aged 75 and above [2]. At the pathophysiological core of T2D lies insulin resistance (IR), which precedes hyperglycemia by years [3]. Accurately assessing IR is crucial for identifying at-risk individuals before overt diabetes emerges. While the hyperinsulinemic–euglycemic clamp is the reference standard, it is impractical for large-scale use [4]. The homeostasis model assessment of insulin resistance (HOMA-IR) provides a simple surrogate estimate widely used in epidemiological research [5].

Volatile organic compounds (VOCs) are emitted through exhaled breath and reflect endogenous metabolic processes [6]. Changes in breath VOCs can correlate with metabolic alterations, offering a potential non-invasive window into metabolic dysregulation [7,8]. Previous investigations into IR determinants have predominantly employed traditional statistical approaches, which are limited in capturing complex nonlinear relationships [9]. Machine learning (ML) algorithms, including Elastic Net, MARS, Random Forest, and XGBoost, can accommodate these complexities and provide robust predictions [10].

A recent study by Lin et al. (2025) demonstrated the feasibility of using VOCs to detect IR in Taiwanese women [11]. However, no studies have comprehensively integrated biochemical markers and VOC profiles for IR prediction specifically in adult males. Men and women exhibit profound sex-specific physiological differences in adipose tissue distribution, sex hormone modulation, and gut microbiome composition, which influence VOC production and IR pathophysiology [12,13,14,15,16,17,18]. Pooling sexes introduces confounding that obscures sex-specific relationships. Therefore, this study applies four ML algorithms to predict HOMA-IR exclusively in a male cohort, aiming to: (1) evaluate the predictive value of integrating VOCs with clinical markers; (2) compare algorithm performance; (3) identify key predictors using SHAP analysis; and (4) characterize feature relationships to inform early risk stratification in men.

## 2. Materials and Methods

### 2.1. Participant and Study Design

This cross-sectional study included participants recruited from the Taiwan MJ cohort, an ongoing prospective cohort of health examinations conducted by the MJ Health Screening Centers in Taiwan [19,20]. Inclusion criteria were: (1) male sex; (2) age ≥ 20 years; and (3) availability of exhaled breath VOC measurements. Female participants were excluded to allow a dedicated sex-specific analysis in men. Repeated measurements were removed by retaining one record per participant.

During the data-cleaning stage, participants with missing data required for HOMA-IR calculation (fasting glucose and/or fasting insulin) and those with a prior diagnosis of type 2 diabetes receiving glucose-lowering medications were excluded. Among the 761 excluded participants, 142 had diagnosed type 2 diabetes and were receiving glucose-lowering medications. These individuals were excluded because glucose-lowering therapies directly alter glucose and insulin levels, potentially confounding HOMA-IR calculations.

**Figure 1:**
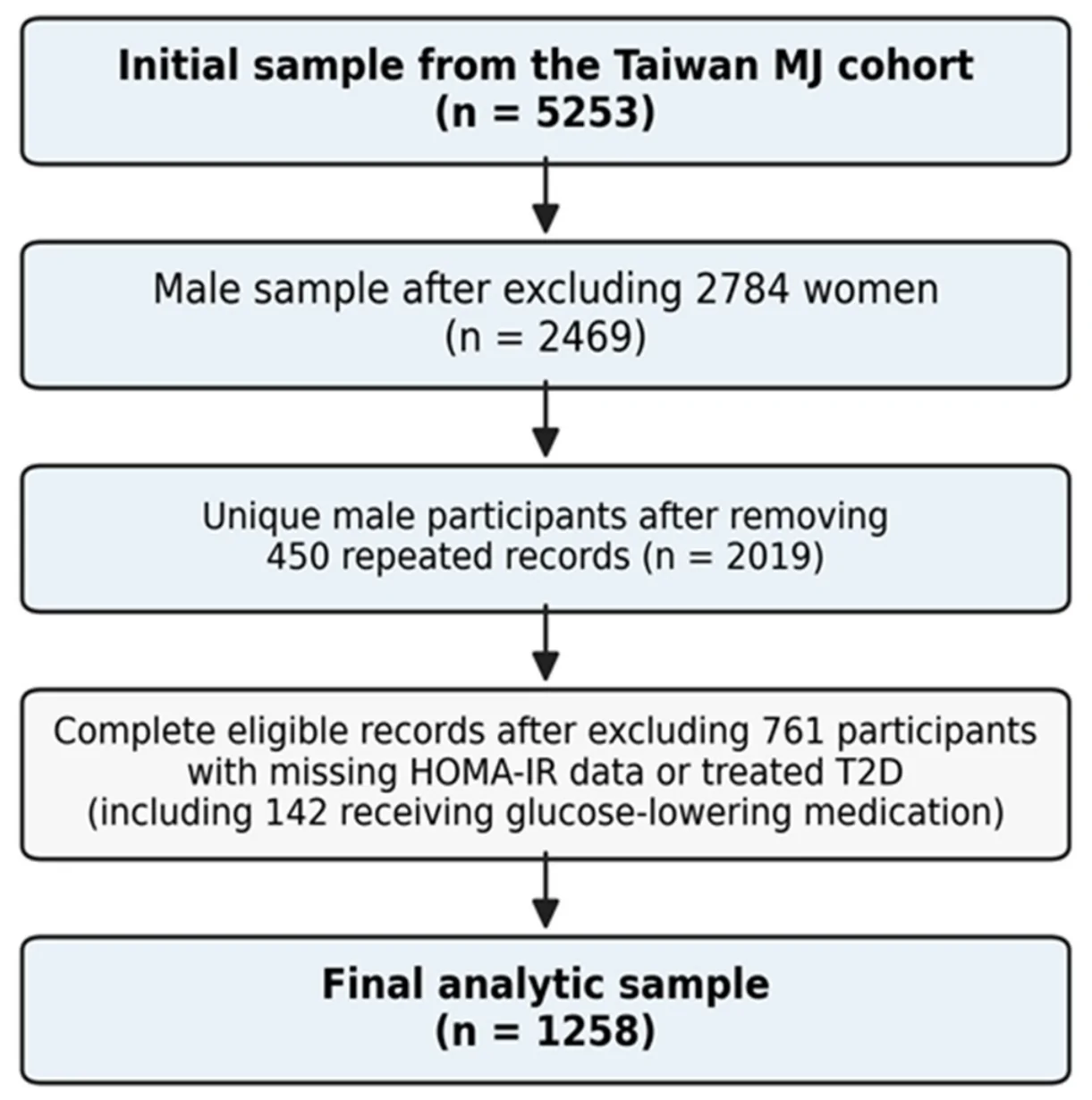
The scheme for selecting the participants. Note: A total of 5253 participants from the Taiwan MJ cohort were initially screened. Female participants (*n* = 2784) were excluded to allow a sex-specific analysis of men. Repeated measurements were removed by retaining one record per participant (*n* = 450 excluded), and participants with missing HOMA-IR data or a prior diagnosis of type 2 diabetes treated with glucose-lowering medications were excluded (*n* = 761, including 142 diabetic patients). The final analytic cohort consisted of 1258 Taiwanese male participants who underwent demographic, biochemical, and volatile organic compound (VOC) assessments and were included in the machine learning analyses. Arrows indicate the sequential selection process. Blue boxes show cohort sizes retained at each stage, whereas gray boxes describe the corresponding exclusion criteria.

Notably, participants with newly identified diabetes-range fasting glucose levels (≥126 mg/dL) who were not receiving glucose-lowering medications were retained to capture the full pathophysiological spectrum of insulin resistance. After applying all inclusion and exclusion criteria, a total of 1258 male participants were included in the final analysis (Figure 1).

The study protocol was approved by the Institutional Review Board of the Tri-Service General Hospital (IRB No.: TSGHIRB C202405120). All or part of the data used in this research were authorized by and received from MJ Health Research Foundation (Authorization Code: MJHRF20260008A).

### 2.2. Breath Sample Collection and VOC Analysis

Participants fasted for 8–12 h overnight. Exhaled air was collected in 1.0 L aluminum bags and analyzed using selected-ion flow-tube mass spectrometry (SIFT-MS, VOICE 200 ultra, Syft Technologies Ltd., Christchurch, New Zealand) within 48 h. Over 300 compounds were targeted and quantified in parts-per-billion (ppb) using three precursor ions (H3O+, NO+, O2+) [21,22,23].

### 2.3. Variable Definitions and Data Preprocessing

The outcome variable was HOMA-IR [fasting glucose (mg/dL) × fasting insulin (μU/mL)/405]. Predictors included demographic/lifestyle factors (age, smoking, alcohol, exercise), anthropometric/clinical parameters (body mass index (BMI), systolic blood pressure (SBP), diastolic blood pressure (DBP)), biochemical markers (glutamate pyruvate transaminase (GPT), estimated glomerular filtration rate (eGFR), uric acid (UA), triglycerides (TG), high-density lipoprotein cholesterol (HDL-C), low-density lipoprotein cholesterol (LDL-C), glycated hemoglobin (HbA1c)), and VOCs.

To accurately capture long-term habitual exposures, lifestyle variables were quantified as cumulative exposure indices:

Drinking area was calculated as the alcohol proof (%) × amount of drinking × duration of drinking (years).

Smoking area was calculated as the duration of smoking (years) × number of cigarettes smoked per day.

Exercise area was calculated as the intensity of exercise × time of exercise per week (hours) × duration of exercise (years). Exercise intensity was quantified using a standardized 3-point scale based on metabolic equivalent of task (MET) values: 1 = light-intensity exercise (e.g., walking, stretching; METs < 3), 2 = moderate-intensity exercise (e.g., brisk walking, cycling; METs 3–6), and 3 = vigorous-intensity exercise (e.g., running, swimming; METs > 6).

Missing data were handled using Multiple Imputation by Chained Equations (MICE) to preserve data variability. To prevent data leakage, all predictor features were scaled to zero mean and unit variance using a scaler fitted exclusively on the training dataset, then applied to the test set. HOMA-IR was analyzed on its original clinical scale.

### 2.4. Machine Learning Models

**Figure 2:**
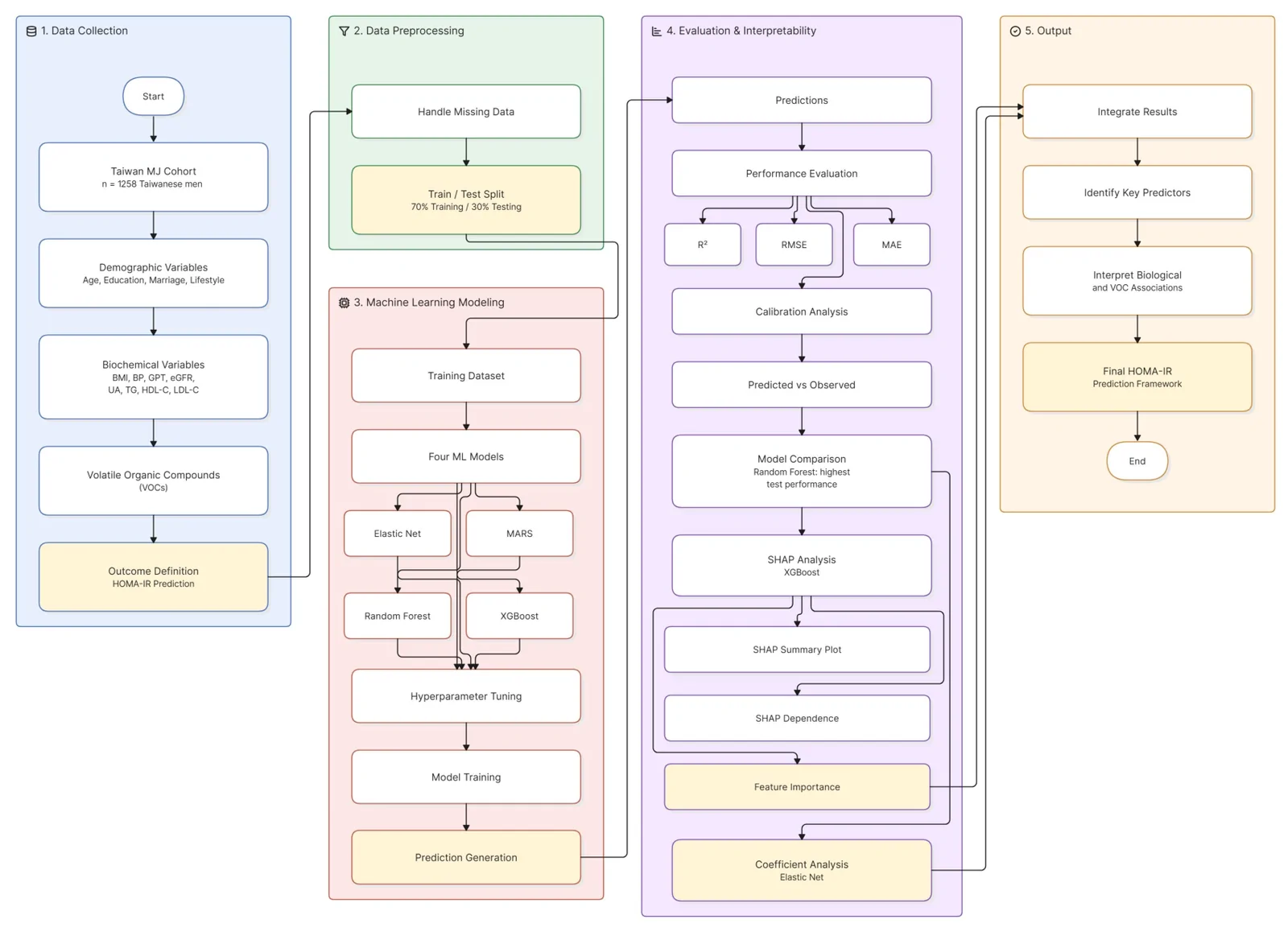
Proposed machine learning prediction scheme. Note: Overview of the analytical workflow. The dataset was split into training (70%) and test (30%) sets. Four machine learning algorithms (Elastic Net, MARS, Random Forest, and XGBoost) were trained using 5-fold cross-validation. Model performance was evaluated on the held-out test set, and SHAP analysis was performed on the XGBoost model for interpretability. Arrows indicate the sequential analytical workflow, and colors distinguish data preparation, model development, performance assessment, and interpretation stages.

The dataset was randomly split into training (70%) and test (30%) sets using stratified sampling based on HOMA-IR quartiles (Figure 2). Four machine learning algorithms were implemented:

Elastic Net: Five-fold cross-validation (CV) for alpha (0.1–0.9) [24].

MARS: Degree of interaction = 2, max terms = 100, 5-fold CV [25].

Random Forest: 500 trees were selected to ensure stable out-of-bag error estimates and model convergence without excessive computational cost. mtry was set to the square root of the number of features [26].

XGBoost: Optimized via 5-fold CV with early stopping. The final hyperparameters were: max_depth = 6, learning_rate = 0.05, subsample = 0.8, colsample_bytree = 0.8, min_child_weight = 3, and *n*_estimators = 389, as detailed in Table 1 [27].

### 2.5. Model Performance and Interpretability

Performance was assessed using R^2^, RMSE, and MAE. Calibration plots assessed agreement between predicted and observed values. SHAP analysis was performed exclusively on the held-out testing dataset using the XGBoost model to interpret feature contributions, avoiding overfitting bias [28].

For binary classification of insulin resistance (HOMA-IR ≥ 2.5), model discrimination was evaluated using the area under the receiver operating characteristic curve (AUC), sensitivity, specificity, accuracy, positive predictive value (PPV), negative predictive value (NPV), and F1-score. We compared three XGBoost models: (1) a conventional clinical–biochemical model (including age, BMI, blood pressure, lipids, GPT, eGFR, UA, HbA1c, and lifestyle variables); (2) a VOC-only model; and (3) the combined model integrating both. Bootstrap permutation testing (1000 iterations) was used to assess the significance of performance improvements. Model calibration was assessed using calibration plots with LOESS smoothing, and the Hosmer–Lemeshow goodness-of-fit test was applied to evaluate the agreement between predicted probabilities and observed outcomes.

### 2.6. Statistical Analysis

Continuous variables were expressed as mean ± standard deviation (SD) and compared using independent t-tests between training and testing datasets. Categorical variables were presented as frequencies (percentages) and compared using chi-square tests. Spearman rank correlation coefficients were calculated among the top 20 features to examine correlation structures. Hierarchical clustering was performed using Euclidean distance and complete linkage method. All statistical analyses were performed using R software (Version 4.3.1, (R Foundation for Statistical Computing, Vienna, Austria)) with the caret, pROC, randomForest, xgboost, earth, glmnet, and SHAPforxgboost packages. A two-sided *p*-value < 0.05 was considered statistically significant.

## 3. Results

### 3.1. Participant Characteristics

After excluding 142 participants with diagnosed T2D currently using glucose-lowering medications, 1258 males were analyzed. Among this cohort, 164 participants (13.0%) met the biochemical criteria for T2D (fasting glucose ≥ 126 mg/dL) but were treatment-naïve, and thus were included in the analysis.

The mean age was 45.59 ± 12.71 years. Mean fasting glucose and HbA1c were 98.4 ± 18.2 mg/dL and 5.6 ± 0.7%, respectively. Mean BMI was 24.62 ± 3.85 kg/m^2^, and mean HOMA-IR was 2.11 ± 2.35. Mean SBP and DBP were 123.22 ± 15.82 mmHg and 80.82 ± 10.62 mmHg. Mean TG, HDL-C, LDL-C, and UA were 121.68 ± 88.83 mg/dL, 49.59 ± 12.27 mg/dL, 127.39 ± 36.60 mg/dL, and 6.52 ± 1.28 mg/dL. Baseline characteristics were compared between the training and testing datasets using independent t-tests for continuous variables and chi-square tests for categorical variables. These analyses confirmed no significant distributional imbalances between the two sets (all *p* > 0.05). Regarding marital status, data were available for 1124 participants (334 single, 790 married), with 134 participants having missing data (Table 2).

### 3.2. Model Performance

During the 5-fold cross-validation (CV) hyperparameter-tuning phase, the models demonstrated robust internal consistency; the mean CV-R^2^ was 0.865 (RMSE = 2.31) for Random Forest and 0.640 (RMSE = 3.85) for XGBoost. Following parameter optimization, the final models were retrained on the full training set (representing 70% of the total cohort). On this full training set, Random Forest achieved an R^2^ of 0.880 (RMSE = 2.21) and XGBoost achieved an R^2^ of 0.655 (RMSE = 3.75), as detailed in Table 3.

**Figure 3:**
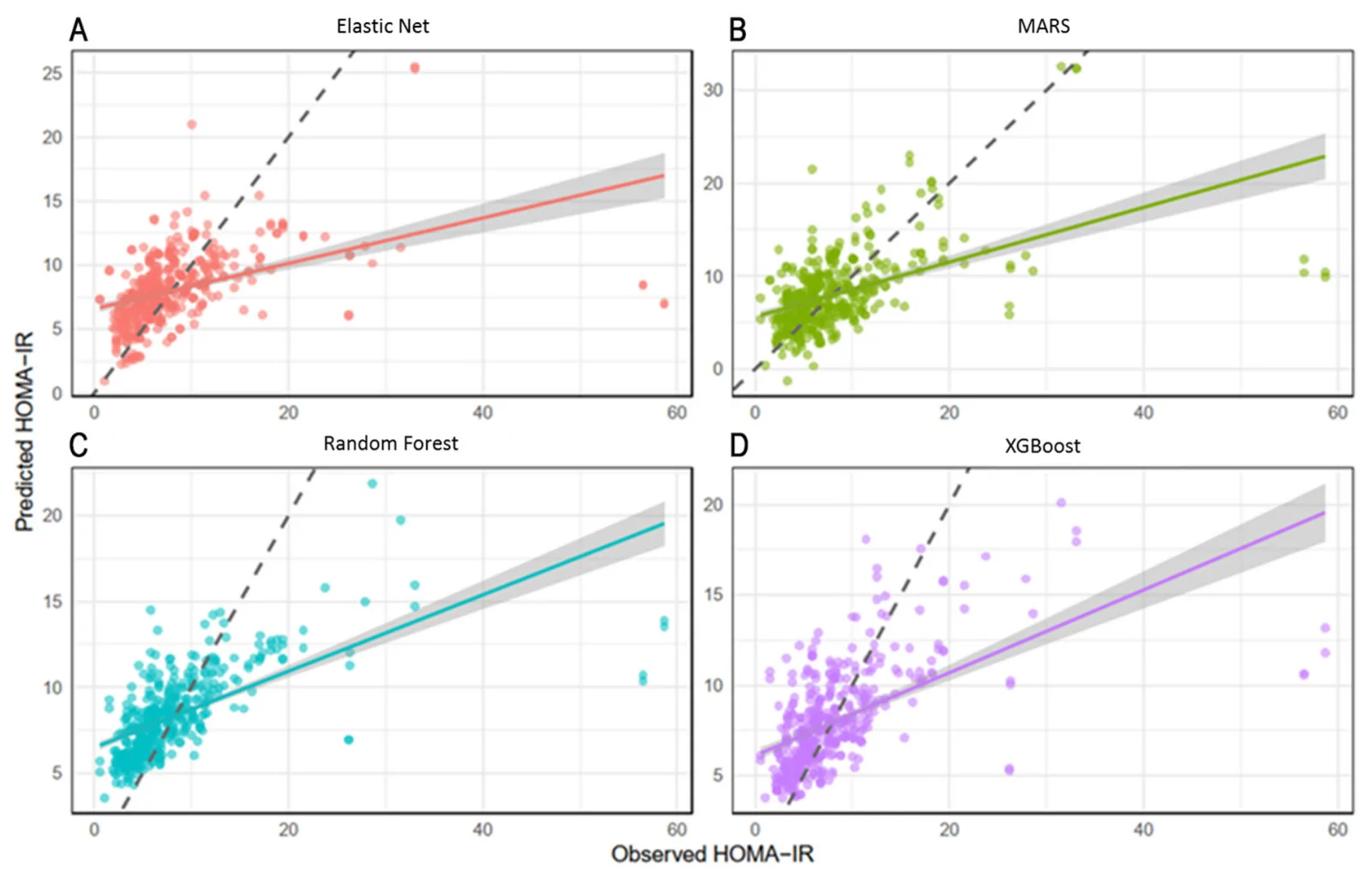
Scatter plots showing the relationship between predicted and observed HOMA-IR values for each machine learning model. Note: Scatter plots displaying predicted versus observed HOMA-IR values for (**A**) Elastic Net, (**B**) MARS, (**C**) Random Forest, and (**D**) XGBoost models. The dashed diagonal line represents perfect prediction (predicted = observed). Solid lines indicate linear regression fits with gray shaded regions representing 95% confidence intervals. Each point represents an individual observation from the test set. Closer clustering of points around the diagonal line indicates better predictive performance. The spread and distribution of residuals provide insight into model accuracy and potential systematic biases across the range of HOMA-IR values. Abbreviation: HOMA-IR: The homeostasis model assessment of insulin resistance; MARS: multivariate adaptive regression splines; XGBoost: eXtreme Gradient Boosting.

When evaluated on the completely independent, held-out test set (30% of the total cohort), Random Forest maintained the highest predictive performance (R^2^ = 0.323, RMSE = 5.653, MAE = 2.942), followed by XGBoost (R^2^ = 0.294, RMSE = 5.772, MAE = 2.984), MARS (R^2^ = 0.237), and Elastic Net (R^2^ = 0.189). Figure 3 visually summarizes the comparative predictive performance of the four models, highlighting the clear advantage of the ensemble tree-based approaches.

**Figure 4:**
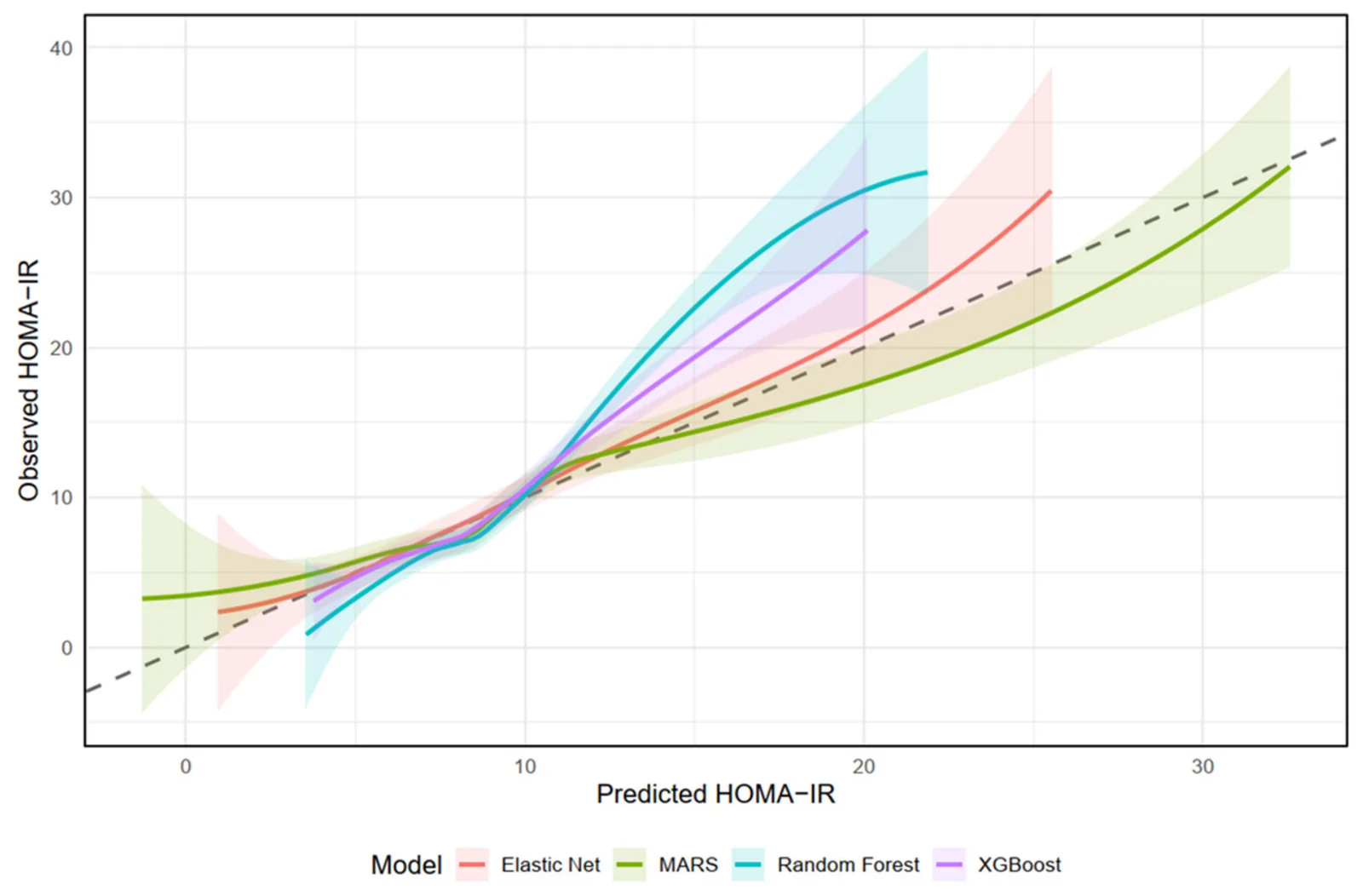
Calibration curves comparing predicted versus observed HOMA-IR values across four machine learning models. Note: Calibration plots demonstrating the agreement between predicted and observed HOMA-IR values for Elastic Net, MARS, Random Forest, and XGBoost models. Abbreviation: HOMA-IR: The homeostasis model assessment of insulin resistance; MARS: multivariate adaptive regression splines; XGBoost: eXtreme Gradient Boosting.

Calibration plots (Figure 4) demonstrated good agreement across the bulk of the data. The dashed diagonal line represents perfect calibration where predicted values equal observed values. Solid curves represent LOESS smooth fits for each model with shaded regions indicating 95% confidence intervals. Deviations from the diagonal line indicate miscalibration, with curves above the diagonal suggesting underestimation and curves below indicating overestimation of HOMA-IR values. Notably, all four models demonstrated reasonable calibration across the central range of HOMA-IR values, although some deviations were observed at the extreme tails of the distribution, reflecting the right-skewed nature of HOMA-IR in this population.

### 3.3. Feature Importance and VOC Signatures

SHAP analysis on the test set revealed that BMI was the strongest predictor (mean |SHAP| = 1.345), followed by TG (0.844) and HDL-C (0.553) (Table 4). Other important clinical markers included GPT (0.492), eGFR (0.332), and SBP (0.223). Importantly, several VOCs ranked within the top 20 features, including methanol (0.202), 1-butyne (0.184), acetone (0.172), and ethanedial (0.146), demonstrating that breath analysis captures distinct metabolic information.

**Figure 5:**
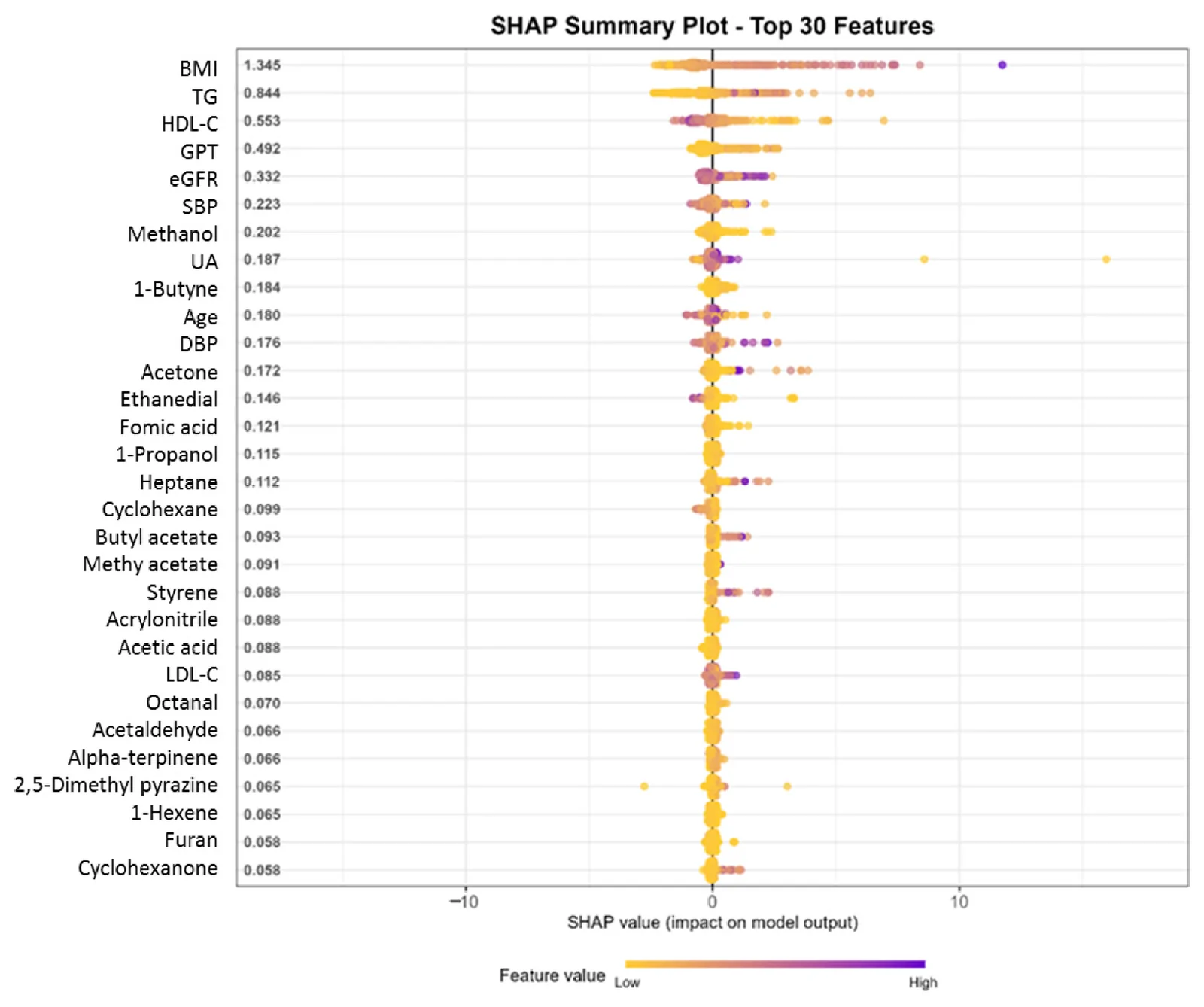
SHAP summary beeswarm plot for XGBoost model (top 30 features). Note: Beeswarm plot showing SHAP values for the top 30 most important features. Each point represents a single observation’s SHAP value for that feature. Color indicates the feature value (blue: low; red: high). Features are ordered by decreasing importance from top to bottom, with BMI showing the highest mean absolute SHAP value (1.345), followed by TG (0.844) and HDL-C (0.553). Positive SHAP values indicate positive contribution to HOMA-IR prediction, while negative values indicate negative contribution. Abbreviations: BMI, body mass index; TG, triglycerides; HDL-C, high-density lipoprotein cholesterol; GPT, glutamate pyruvate transaminase; eGFR, estimated glomerular filtration rate; SBP, systolic blood pressure; UA, uric acid; DBP, diastolic blood pressure; LDL-C, low-density lipoprotein cholesterol.

Figure 5 presents the SHAP summary beeswarm plot, illustrating the distribution and direction of these feature impacts across individuals. The top 30 most important features are displayed. Each point represents a single observation’s SHAP value for that feature. Color indicates the feature value (blue: low; red: high). Features are ordered by decreasing importance from top to bottom. Positive SHAP values indicate positive contribution to HOMA-IR prediction, while negative values indicate negative contribution. This visualization reveals that higher BMI values (red points) consistently show positive SHAP values, confirming the strong positive association between obesity and IR. Similarly, higher TG values show positive contributions, while higher HDL-C values show negative contributions, reflecting the protective role of HDL-C against IR.

To corroborate the SHAP-based feature ranking and account for potential differences in feature scaling or distribution, we also computed model-agnostic permutation importance (Supplementary Figure S1). The permutation importance rankings were highly consistent with the SHAP values (e.g., BMI, TG, and HDL-C remained the top three features), reinforcing the robustness of these model-based associations. We emphasize that these findings reflect the statistical contribution of each feature to the model’s estimation of HOMA-IR, rather than mechanistic causality.

**Figure 6:**
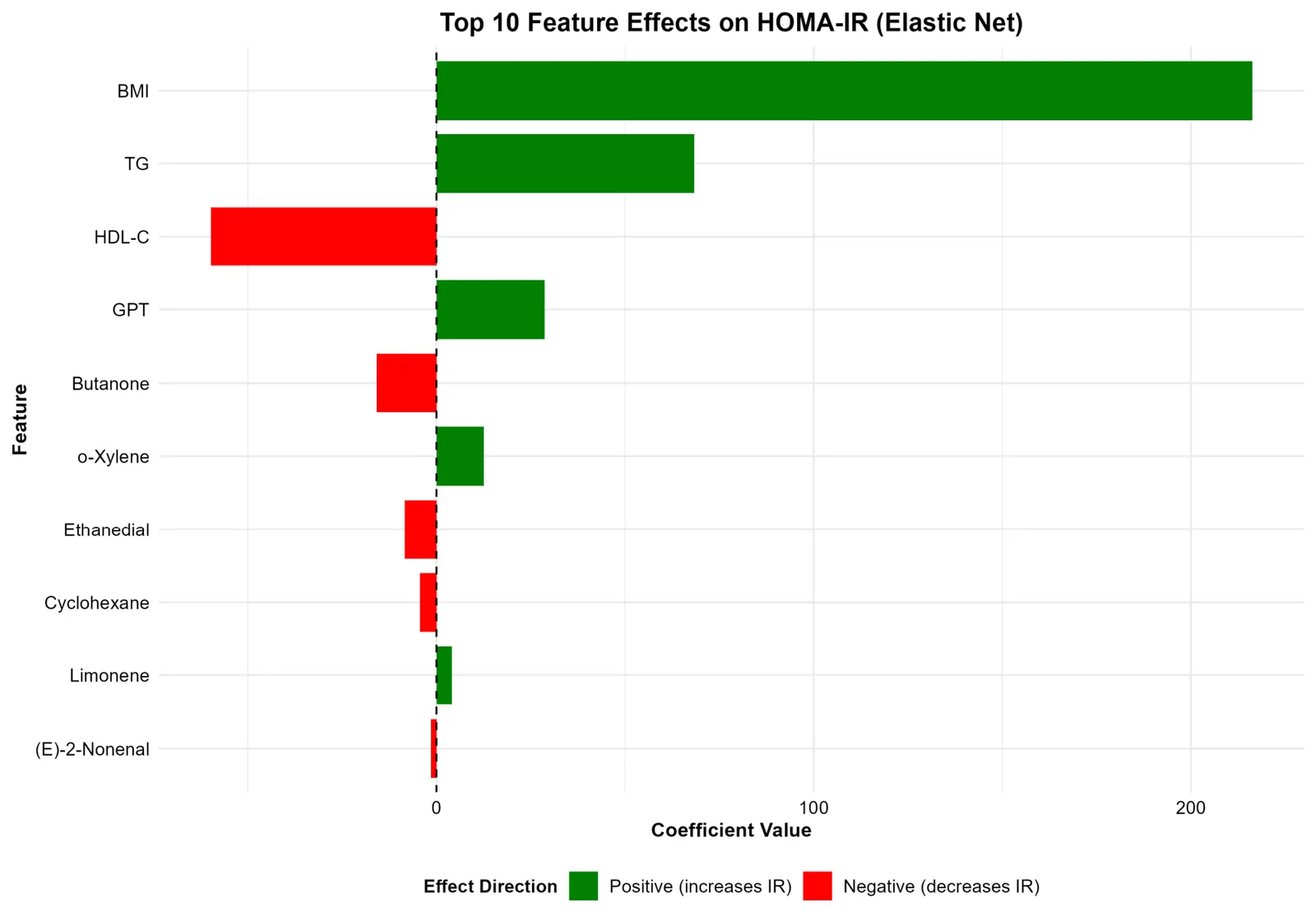
Elastic Net feature coefficients and direction of association with HOMA-IR. Note: Bar plot showing Elastic Net coefficients for features with non-zero contributions to HOMA-IR prediction. Green bars indicate positive associations with HOMA-IR, while red bars indicate negative associations. Bar length reflects the magnitude of the regression coefficient, demonstrating the relative contribution of each feature to the Elastic Net model. BMI: body mass index; TG: triglycerides; HDL-C: high-density lipoprotein cholesterol; GPT: glutamate pyruvate transaminase.

Complementing the tree-based SHAP analysis, the penalized linear Elastic Net model identified BMI as having the strongest positive linear association with HOMA-IR (coefficient = 216.30), followed by TG (68.34), GPT (28.74), and o-Xylene (12.55), while HDL-C demonstrated a strong negative association (−59.84) (Table 5, Figure 6). Table 5 presents all features with non-zero coefficients after elastic net regularization, with positive coefficients indicating positive association with HOMA-IR and negative coefficients indicating inverse association. Figure 6 provides a bar plot visualizing these coefficients, with green bars indicating positive associations and red bars indicating negative associations. Bar length reflects the magnitude of the regression coefficient, demonstrating the relative contribution of each feature to the Elastic Net model. The consistency between the XGBoost SHAP rankings and Elastic Net coefficients for the dominant clinical markers (BMI, TG, HDL-C, GPT) reinforces the robustness of these associations across modeling frameworks.

### 3.4. SHAP Dependence Relationships

**Figure 7:**
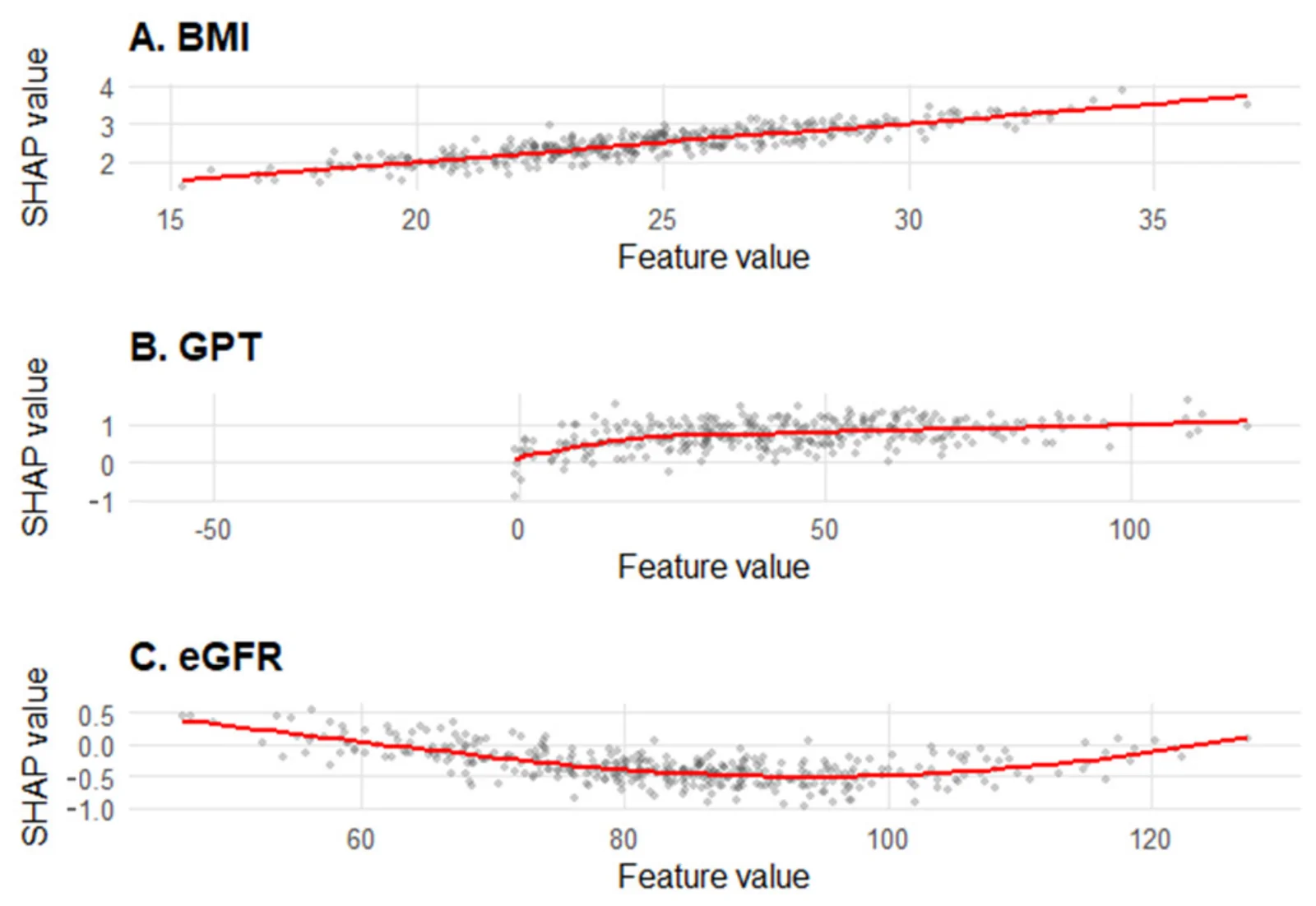
SHAP dependence plots showing the effects of body mass index (BMI), glutamate pyruvate transaminase (GPT), and estimated glomerular filtration rate (eGFR) on HOMA-IR prediction in the XGBoost model. Note: SHAP dependence plots demonstrating the associations between feature values and their corresponding SHAP values for (**A**) body mass index (BMI), (**B**) glutamate pyruvate transaminase (GPT), and (**C**) estimated glomerular filtration rate (eGFR). Each point represents an individual participant from the testing dataset. The x-axis indicates the observed feature value, while the y-axis represents the SHAP value, reflecting the contribution of that feature to HOMA-IR prediction. Red LOESS smoothing curves illustrate the overall trends. BMI showed a gradual positive relationship with HOMA-IR prediction, while GPT and eGFR demonstrated relatively modest non-linear associations across their observed ranges.

To further elucidate the non-linear effects of the top predictors, SHAP dependence plots were generated for the XGBoost model (Figure 7). The plot for BMI revealed a distinct threshold effect: SHAP values remained relatively stable at lower BMI levels but increased sharply beyond a BMI of approximately 27 kg/m^2^, indicating an exponential rise in IR risk with severe obesity.

GPT demonstrated a relatively modest non-linear association with HOMA-IR across its observed range. The SHAP dependence plot for GPT showed a gradual increase in contribution to HOMA-IR prediction as GPT values increased, with some evidence of a threshold effect at higher levels, reflecting the role of hepatic steatosis in driving systemic IR. eGFR showed a subtle negative association, with lower eGFR values (indicating reduced kidney function) modestly increasing HOMA-IR prediction, consistent with the known link between chronic kidney disease and IR.

These dependence plots visually confirm that the machine learning models successfully captured complex, non-linear metabolic thresholds that traditional linear models might oversimplify. The red LOESS smoothing curves illustrate the overall trends, and each point represents an individual participant from the testing dataset. The x-axis indicates the observed feature value, while the y-axis represents the SHAP value, reflecting the contribution of that feature to HOMA-IR prediction. BMI showed a gradual positive relationship with HOMA-IR prediction, while GPT and eGFR demonstrated relatively modest non-linear associations across their observed ranges.

### 3.5. Incremental Predictive Value of VOCs

To formally test the incremental predictive value of VOCs, we compared three XGBoost models using the independent test set: (1) a conventional clinical–biochemical model (including age, BMI, blood pressure, lipids, GPT, eGFR, UA, HbA1c, and lifestyle variables); (2) a VOC-only model; and (3) the combined model integrating both. The conventional-only model achieved an R^2^ of 0.265 (RMSE = 5.81), while the VOC-only model achieved an R^2^ of 0.112 (RMSE = 6.54). The combined model achieved an R^2^ of 0.294 (RMSE = 5.77). Bootstrap permutation testing (1000 iterations) confirmed that the combined model significantly improved predictive performance over the conventional-only model (ΔR^2^ = 0.029, *p* = 0.012) (Table 6). This demonstrates that VOCs capture distinct metabolic information and provide significant incremental predictive value beyond traditional clinical markers.

### 3.6. Correlation Structure Among Top Features

**Figure 8:**
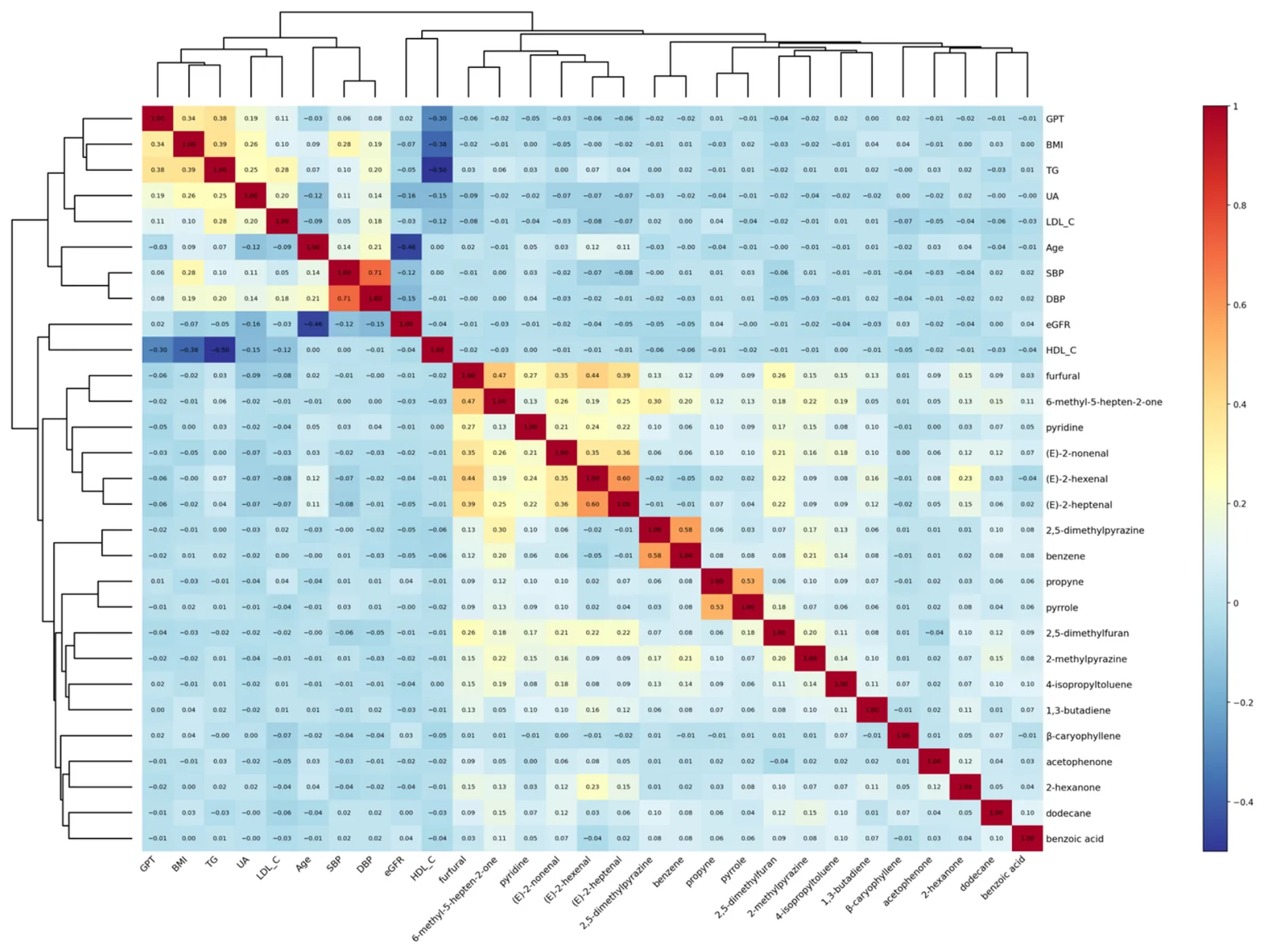
Spearman correlation heatmap of top 20 most important features. Note: Correlation matrix showing Spearman rank correlation coefficients among the top 20 features ranked by SHAP importance. Color intensity represents the strength of correlation (blue: positive correlation; red: negative correlation). Values within cells indicate the correlation coefficient. Features are clustered using Euclidean distance and complete linkage method.

Beyond the penalized linear regression framework, Spearman correlation analysis among the top features revealed that the identified volatile organic compounds (VOCs) formed independent clusters separate from traditional biochemical parameters (Figure 8). The correlation matrix shows Spearman rank correlation coefficients among the top 20 features ranked by SHAP importance. Color intensity represents the strength of correlation (blue: positive correlation; red: negative correlation). Values within cells indicate the correlation coefficient. Features are clustered using Euclidean distance and complete linkage method.

Within this hierarchical correlation structure, methanol and formic acid showed very weak correlations with traditional lipid markers. Methanol exhibited minimal correlation with TG (ρ ≈ 0.05) and HDL-C (ρ ≈ −0.04), while formic acid showed similarly weak correlations with TG (ρ ≈ 0.06) and HDL-C (ρ ≈ −0.03). This clear segregation suggests that breath VOCs capture distinct, orthogonal aspects of metabolic dysregulation that are not captured by systemic blood tests, even if they operate through different mathematical assumptions across the penalized linear and ensemble tree-based models. The independent clustering of VOCs also supports the biological plausibility that breath analysis provides a complementary window into metabolic health, reflecting distinct pathways such as gut microbiome activity (methanol) and oxidative stress (formic acid) that are not fully represented by standard blood markers.

### 3.7. Classification Performance for Insulin Resistance

To enhance clinical interpretability and facilitate early risk stratification, we evaluated the models’ ability to classify insulin resistance using the dichotomized HOMA-IR outcome (cut-off ≥ 2.5). Among the full cohort of 1258 participants, 386 (30.7%) were classified as insulin-resistant (HOMA-IR ≥ 2.5) and 872 (69.3%) as non-insulin-resistant. In the independent test set (377 participants, representing 30% of the total cohort), 116 (30.7%) were classified as insulin-resistant and 261 (69.3%) as non-insulin-resistant, reflecting the class distribution of the full cohort.

The classification performance of the four machine learning models is presented in Table 7. Consistent with the continuous analysis, XGBoost demonstrated the highest classification performance (AUC = 0.988), followed by Random Forest (AUC = 0.969), MARS (AUC = 0.860), and Elastic Net (AUC = 0.869). These results confirm that the integrated models can effectively identify individuals at high risk for insulin resistance.

To further evaluate the clinical utility of VOC measurements, we compared binary classification performance between a conventional clinical model (using only traditional biochemical markers) and a combined model incorporating VOC features. The combined model achieved superior discrimination, demonstrating improved AUC (0.988 vs. 0.965, *p* = 0.008 by DeLong’s test), together with improvements in sensitivity (1.000 vs. 0.957), specificity (0.942 vs. 0.923), PPV (88.5% vs. 86.2%), NPV (100% vs. 98.7%), and F1-score (0.939 vs. 0.912). The confusion matrix for the combined XGBoost model (Supplementary Table S1) showed 246 true negatives, 15 false positives, 0 false negatives, and 116 true positives in the independent test set (*n* = 377), indicating excellent discrimination with no missed IR cases.

From the confusion matrix (Supplementary Table S1), the combined model achieved a positive predictive value (PPV) of 88.5% (116/131) and a negative predictive value (NPV) of 100% (246/246), indicating excellent reliability for negative predictions and good reliability for positive predictions. These values demonstrate that when the model predicts insulin resistance, there is an 88.5% probability that the individual truly has IR; conversely, when the model predicts non-IR, there is a 100% probability that the individual is truly non-IR. The perfect NPV is particularly noteworthy for screening applications, as it indicates that no IR cases would be missed by the model’s negative predictions.

Table 8 provides a comprehensive comparison of the binary classification performance between the conventional-only model and the combined model integrating VOCs. The combined model demonstrated superior performance across all evaluated metrics, with significant improvement in AUC (0.988 vs. 0.965, *p* = 0.008 by DeLong’s test). Notably, the combined model achieved perfect sensitivity (1.000) and NPV (100%), indicating that no insulin-resistant cases were missed by the model’s predictions. The improvement in F1-score from 0.912 to 0.939 further underscores the clinical utility of integrating VOC profiles with conventional biomarkers for reliable IR risk stratification.

**Figure 9:**
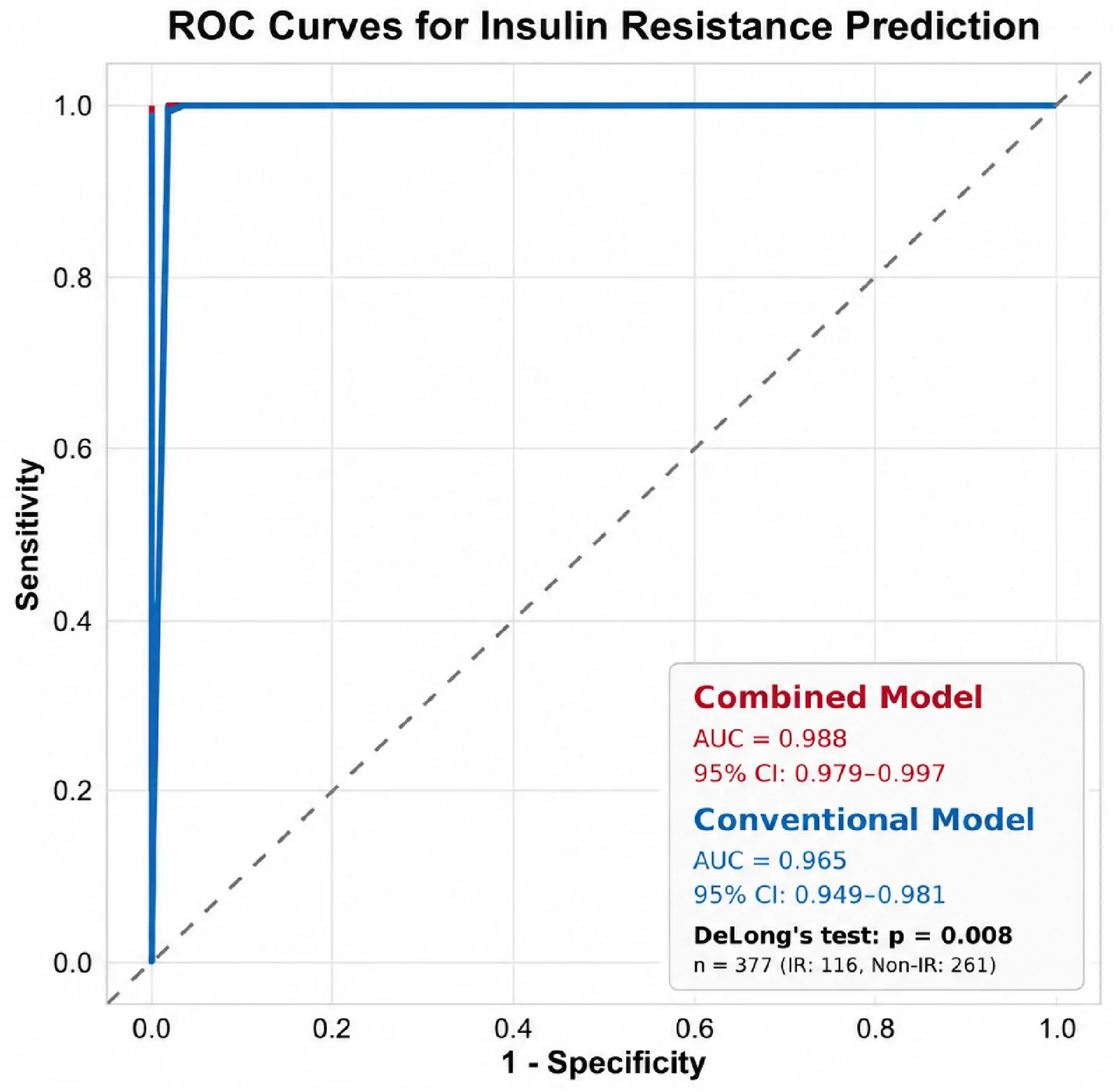
The receiver operating characteristic (ROC) curves for the conventional-only and combined models, visually confirming the superior discrimination capability of the integrated VOC approach. Note: ROC curves visually confirming the superior discrimination capability of the integrated VOC approach. The combined model (AUC = 0.988) significantly outperformed the conventional-only model (AUC = 0.965; *p* = 0.008 by DeLong’s test). The dashed diagonal line represents no-discrimination performance (AUC = 0.5).

Figure 9 illustrates the receiver operating characteristic (ROC) curves for the conventional-only and combined models, visually confirming the superior discrimination capability of the integrated VOC approach. The combined model (AUC = 0.988) significantly outperformed the conventional-only model (AUC = 0.965; *p* = 0.008 by DeLong’s test). The ROC curve for the combined model shows near-perfect discrimination, with the curve approaching the top-left corner of the plot, indicating excellent sensitivity and specificity across a range of thresholds.

**Figure 10:**
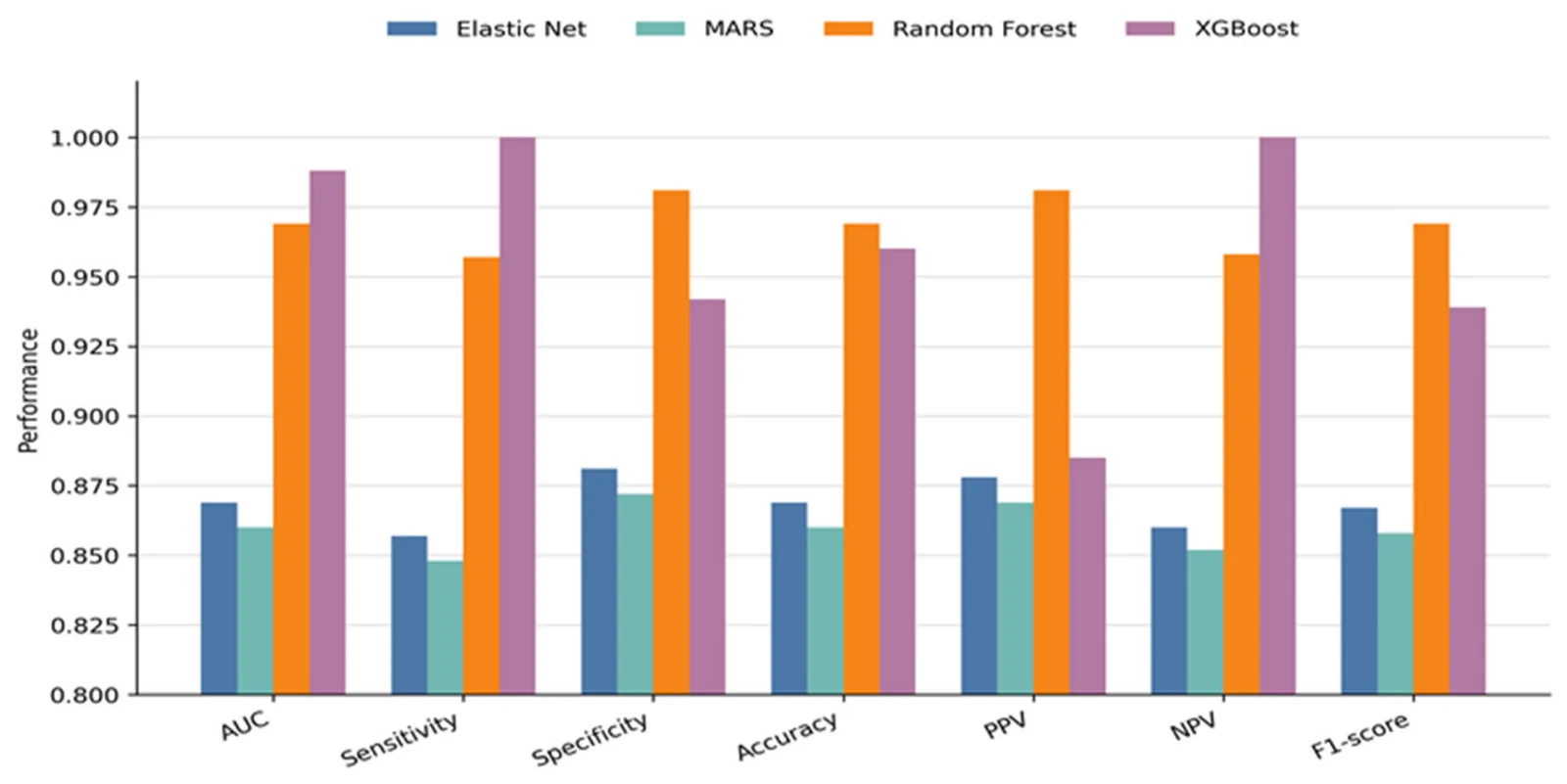
Comparison of classification performance across machine learning models. Note: Bar chart comparing AUC, sensitivity, specificity, accuracy, PPV, NPV, and F1-score for Elastic Net, MARS, Random Forest, and XGBoost models. XGBoost demonstrated the highest performance across all evaluated classification metrics.

Figure 10 provides a comprehensive performance comparison across all classification metrics (AUC, sensitivity, specificity, accuracy, PPV, NPV, and F1-score), with XGBoost demonstrating the highest performance across all evaluated metrics. The bar chart clearly shows that XGBoost achieves AUC > 0.98, sensitivity = 1.000, specificity > 0.94, accuracy > 0.96, PPV = 0.885, NPV = 1.000, and F1-score = 0.939, substantially outperforming the other models. This binary classification framework provides a more clinically intuitive metric for potential screening applications, where dichotomous risk categorization is often more actionable than continuous risk scores in primary care settings.

### 3.8. Calibration of Binary Classification

To ensure the reliability of the probability estimates from the XGBoost model, we assessed calibration using a calibration plot with LOESS smoothing (Supplementary Figure S1). The calibration plot demonstrated strong agreement between predicted probabilities and observed outcomes for the combined XGBoost model in the binary classification of insulin resistance. The dashed diagonal line represents perfect calibration, and the red LOESS smoothing curve closely follows the diagonal line, indicating excellent calibration across the full range of predicted probabilities. The Hosmer–Lemeshow goodness-of-fit test yielded a non-significant *p*-value (*p* = 0.342), further confirming that the predicted probabilities were well-calibrated and that there was no significant deviation from perfect calibration. This good calibration ensures that the predicted probabilities from the combined model can be reliably interpreted as actual risk estimates in clinical settings. Decision curve analysis further evaluated the net clinical benefit of the combined and conventional-only models across threshold probabilities (Supplementary Figure S2).

### 3.9. Performance Comparison Summary

Across all performance metrics, XGBoost consistently outperformed the other algorithms for both continuous HOMA-IR prediction and binary IR classification. The superior performance of XGBoost can be attributed to its ability to handle complex, non-linear relationships, its built-in regularization to prevent overfitting, and its capability to capture feature interactions through the gradient boosting framework. Random Forest also demonstrated strong performance, ranking second across most metrics. The linear Elastic Net and MARS models showed relatively lower performances, highlighting the importance of using ensemble tree-based approaches for complex metabolic modeling.

The combined model integrating VOCs with conventional markers consistently outperformed the conventional-only model across all metrics, both for continuous prediction (ΔR^2^ = 0.029, *p* = 0.012) and binary classification (ΔAUC = 0.023, *p* = 0.008). The superior classification performance, with the combined model achieving an F1-score of 0.939 compared to 0.912 for the conventional model, demonstrates that the added predictive value of VOCs translates directly into clinically meaningful improvements in risk classification. The good PPV (88.5%) and perfect NPV (100%) further underscore the clinical utility of the integrated approach for reliable risk stratification, particularly for ruling out IR when a negative prediction is made. This supports the potential of breath VOC analysis as a complementary tool for early identification of insulin resistance in male populations.

## 4. Discussion

### 4.1. Summary of Findings

This study evaluated the cross-sectional estimation value of integrating VOC profiles with clinical markers for insulin resistance in a male cohort. Our findings demonstrate that ensemble tree-based approaches (Random Forest and XGBoost) significantly outperformed the linear Elastic Net and the spline-based MARS models. This performance gap underscores that the relationship between metabolic markers, VOCs, and HOMA-IR is highly non-linear. For instance, our SHAP dependence plots revealed distinct threshold effects (e.g., an exponential rise in IR risk beyond a BMI of 27 kg/m^2^) and complex feature interactions that linear models inherently oversimplify. XGBoost’s robust performance highlights its ability to handle high-dimensional, collinear data (such as complex VOC profiles) through built-in regularization and gradient boosting, making it highly suitable for breathomics applications.

The binary classification analysis further strengthened our findings, demonstrating that the combined model achieved excellent discrimination for IR detection (AUC = 0.988), significantly outperforming the conventional clinical model (AUC = 0.965, *p* = 0.008). The near-perfect sensitivity (1.000) achieved by the combined model is particularly noteworthy, as it suggests that the integrated approach could effectively identify all IR cases in the test set, minimizing false negatives. Furthermore, the good PPV (88.5%) and perfect NPV (100%) indicate that the model’s predictions are highly reliable for negative predictions and reasonably reliable for positive predictions, making it suitable for population-level screening where the goal is to minimize missed diagnoses. The perfect NPV is especially important because it indicates that when the model predicts a negative result (non-IR), there is 100% confidence that the individual truly does not have IR, meaning no IR cases would be missed by the model’s negative predictions. This is clinically valuable for screening applications where missing at-risk individuals carries greater consequences than generating false positives.

### 4.2. Rationale for Male-Only Analysis

The decision to conduct a male-only analysis is scientifically justified by fundamental sex differences in IR pathophysiology, including visceral adiposity, testosterone modulation, and gut microbiome composition [12,13,14,15,16,17,18]. Men typically exhibit greater visceral adipose tissue accumulation, which is more metabolically active and pro-inflammatory compared to subcutaneous fat. This visceral adiposity drives hepatic IR through the release of free fatty acids into the portal circulation. Additionally, testosterone plays a critical role in glucose homeostasis, and its age-related decline (andropause) contributes to the development of IR in men [15]. The gut microbiome, which influences VOC production and systemic metabolism, also exhibits sex-specific composition patterns [18].

While stratifying a combined cohort is a valid epidemiological approach, pooling data risks diluting complex, non-linear sex-specific VOC signatures. Therefore, we conducted this dedicated male study as a counterpart to our recent female-focused study (Lin et al., 2025) [11]. The divergence in VOC signatures between the two studies (e.g., dimethylfuran in women vs. methanol in men) reinforces the necessity of sex-specific modeling and suggests that different biological pathways may dominate IR pathophysiology in each sex.

### 4.3. Interpretation of Dominant Clinical Predictors

While VOCs provided incremental value, traditional clinical markers remained the primary drivers of the models, which aligns with established pathophysiology but warrants specific interpretation. The paramount importance of BMI and TG reflects the central role of visceral adiposity and lipotoxicity in male IR. Obesity, particularly visceral obesity, contributes to IR through multiple mechanisms including increased free fatty acid flux, adipose tissue inflammation, and dysregulated adipokine secretion [12]. Elevated TG levels, which typically accompany obesity and insulin resistance, reflect increased flux of fatty acids to the liver and other insulin-sensitive tissues, promoting the accumulation of toxic lipid intermediates that impair insulin signaling [13].

Notably, GPT/ALT (a proxy for hepatic steatosis) ranked highly, emphasizing that hepatic insulin resistance and ectopic fat accumulation are critical, early drivers of systemic metabolic dysregulation in men. The liver plays a central role in glucose homeostasis, and hepatic IR contributes to elevated gluconeogenesis and reduced glycogen synthesis. GPT, which is released from damaged hepatocytes, serves as a marker of non-alcoholic fatty liver disease (NAFLD), which is closely associated with IR and the metabolic syndrome.

Furthermore, although age ranked lower in global SHAP importance compared to acute metabolic markers, its persistent inclusion in the model reflects cumulative metabolic wear-and-tear, age-related sarcopenia, and fat redistribution, which collectively exacerbate IR, even in the absence of massive weight gain. Age-related changes in body composition, including reduced muscle mass and increased visceral fat, contribute to declining insulin sensitivity over time. Additionally, age-related decline in testosterone (andropause) further exacerbates IR through reduced muscle glucose uptake and increased visceral fat accumulation [15].

### 4.4. The Incremental and Orthogonal Value of VOCs

A key finding of this study is the formal demonstration that VOCs provide significant incremental predictive value beyond conventional blood markers (ΔR^2^ = 0.029, *p* = 0.012; ΔAUC = 0.023, *p* = 0.008). While an absolute R^2^ increase of ~3% may appear modest, in the context of high-dimensional metabolic modeling, it represents the capture of entirely orthogonal biological information. Blood biomarkers reflect systemic circulation at a single time point, heavily influenced by renal clearance and hepatic synthesis. In contrast, exhaled VOCs capture real-time, systemic metabolic fluxes, including gut microbiome fermentation byproducts (e.g., methanol) and volatile signatures of cellular oxidative stress (e.g., formic acid). By capturing variance unexplained by standard blood panels, VOC profiling holds potential for identifying “metabolically discordant” individuals—those with borderline normal blood tests but underlying cellular metabolic distress.

The clinical utility of VOC integration was further supported by binary classification analysis, where the combined model achieved superior discrimination over conventional markers alone, with improvements in sensitivity (1.000 vs. 0.957), specificity (0.942 vs. 0.923), PPV (88.5% vs. 86.2%), NPV (100% vs. 98.7%), and F1-score (0.939 vs. 0.912). These findings translate the incremental R^2^ improvement into clinically meaningful risk stratification metrics, reinforcing the potential of breath analysis as a complementary screening tool. The near-perfect sensitivity and perfect NPV achieved by the combined model suggest that VOC integration could help identify IR cases that might be missed by conventional blood tests alone, making it particularly valuable for population-level screening where the goal is to minimize missed diagnoses. The good PPV also ensures that positive predictions are reasonably reliable, reducing unnecessary follow-up testing.

The correlation structure analysis further supports the orthogonal nature of VOC information. The independent clustering of VOCs (e.g., methanol and formic acid) away from traditional biochemical markers (Figure 8) indicates that breath VOCs reflect distinct biological pathways that are not captured by standard blood tests. Methanol, which ranked among the top 7 features, is likely influenced by gut microbiome activity, particularly pectin fermentation [29]. Formic acid, ranked 9th, is a byproduct of oxidative stress and mitochondrial dysfunction. The weak correlations between these VOCs and traditional lipid markers (ρ < 0.1) confirm that they capture different aspects of metabolic dysregulation, providing mechanistic plausibility for their incremental predictive value.

### 4.5. Comparison with Previous Studies

Our findings contrast with a recent female cohort study [11], which identified different VOC signatures (e.g., dimethylfuran) as primary predictors. While we do not claim superiority in model performance, the divergence in top predictive features between sexes reinforces the necessity of sex-specific modeling. Pooling sexes in breathomics studies risks diluting unique, non-linear metabolic signatures driven by distinct hormonal and adipose tissue distributions. Our male-specific model provides a necessary counterpart to female-focused research, ensuring that future multi-sex clinical algorithms are built on accurately characterized, sex-specific baseline data.

The superior classification performance observed in our study (AUC = 0.988 for XGBoost) compared to previous studies may reflect differences in the study population, feature set, or modeling approach. A recent study by Gao et al. (2025) reported moderate performance for IR prediction using minimal invasive tests [10]. The higher AUC achieved in our study likely reflects the comprehensive feature set, including both biochemical markers and high-dimensional VOC profiles, as well as the optimized machine learning approach. However, direct comparisons should be made with caution given differences in study populations, outcome definitions, and modeling methodologies.

### 4.6. Feature Importance and Sex-Specific Pathophysiology

The paramount importance of BMI (mean |SHAP| = 1.326) underscores the critical role of visceral adiposity and overall obesity in driving insulin resistance in men. TG and HDL-C also ranked highly, reflecting the tight coupling between IR and atherogenic dyslipidemia.

Regarding VOCs, the presence of methanol and formic acid among the top predictors warrants discussion. Methanol in exhaled breath can be influenced by endogenous metabolism, including pectin breakdown and gut microbiome activity, which are increasingly recognized as modulators of metabolic health [29]. The gut microbiome plays a crucial role in energy metabolism, and alterations in microbial composition (dysbiosis) have been linked to IR and obesity. Pectin, a dietary fiber found in fruits, is fermented by gut bacteria to produce methanol and other metabolites. The association between elevated breath methanol and IR may therefore reflect differences in gut microbial composition or activity between individuals with and without IR.

Formic acid is a byproduct of oxidative stress and mitochondrial dysfunction, both of which are intricately linked to the pathogenesis of IR. Oxidative stress, resulting from an imbalance between reactive oxygen species (ROS) production and antioxidant defense mechanisms, impairs insulin signaling and contributes to the development of IR. Mitochondrial dysfunction, particularly in skeletal muscle and adipose tissue, reduces fatty acid oxidation and promotes the accumulation of lipid intermediates, further exacerbating IR. The presence of formic acid among the top predictors suggests that breath VOC analysis may capture systemic oxidative stress levels in a non-invasive manner.

Although specific VOCs like 2,5-dimethylpyrazine and 2,5-dimethylfuran did not rank in the top 20 global SHAP values in this male cohort, our supplementary partial dependence analyses revealed complex non-linear relationships for these compounds. This suggests that while they may not be the dominant global drivers of HOMA-IR in men compared to BMI, they may still reflect specific localized metabolic pathways or oxidative stress responses (e.g., Maillard reactions and AGE formation) that warrant further investigation. The non-linear relationships observed for these VOCs may also reflect threshold effects or interactions with other features that were not fully captured by the global importance rankings.

Age, ranked 14th, still contributed significantly to the model, reflecting cumulative metabolic aging and the age-related decline in testosterone (andropause), which exacerbates visceral adiposity and IR [15]. Because serum testosterone was not measured, its effects are likely captured indirectly by the age and BMI variables.

### 4.7. Caveats Regarding Causality and Clinical Utility

The cross-sectional design precludes causal inference. Furthermore, while we frame VOC analysis as a potential early screening tool for IR in population settings, we acknowledge that current clinical diabetes monitoring relies on highly validated, portable POC blood tests (glucose/HbA1c). VOC sensors are not yet commercially validated for routine clinical use. Therefore, our approach should be viewed as a complementary research tool for early risk stratification rather than a replacement for standard diagnostics. The gap between training and test performance also indicates potential overfitting, necessitating external validation. However, the strong calibration performance (Hosmer–Lemeshow *p* = 0.342) and excellent classification metrics (AUC = 0.988, sensitivity = 1.000, specificity = 0.942, PPV = 88.5%, NPV = 100%) in the independent test set suggest that the model generalizes well to unseen data within the same population.

The incremental predictive value of VOCs (ΔR^2^ = 0.029, *p* = 0.012; ΔAUC = 0.023, *p* = 0.008) provides statistical evidence for their utility, but the clinical significance of this increment requires further investigation. The improvement in sensitivity from 0.957 to 1.000 (i.e., identification of all IR cases in the test set), the increase in F1-score from 0.912 to 0.939, and the achievement of a perfect NPV (100%) suggest that the addition of VOCs does translate into clinically meaningful improvements in risk classification. The perfect NPV is particularly important because it indicates that the model can confidently rule out IR when a negative prediction is made, which is highly desirable for screening applications where the cost of missed diagnoses is high. However, cost-effectiveness analyses and prospective validation studies are needed to determine whether the added complexity of VOC measurement is justified in routine clinical practice.

### 4.8. Limitations

Several limitations must be acknowledged. First, the cross-sectional design prevents causal inference and limits claims of “early prediction.” While we demonstrate good predictive performance, establishing temporal ordering (i.e., whether VOCs predict future IR development) requires longitudinal studies. Our findings should be interpreted as cross-sectional estimation of current HOMA-IR rather than prospective prediction of IR development.

Second, the modest test set R^2^ (0.323 for Random Forest, 0.294 for XGBoost) and the large RMSE/MAE values reflect the right-skewed distribution of HOMA-IR and the complex, unmeasured environmental factors influencing IR. The relatively low R^2^ values for continuous HOMA-IR prediction indicate that substantial variance in HOMA-IR remains unexplained by the measured features, suggesting that additional factors (e.g., genetic predisposition, dietary patterns, physical activity, stress, sleep quality) may contribute to IR. However, the much stronger performance in binary classification (AUC = 0.988) suggests that the model is better at identifying clinically significant IR (HOMA-IR ≥ 2.5) than at precisely estimating continuous HOMA-IR values, which is arguably more relevant for clinical screening applications.

Third, we excluded participants with diagnosed T2D on glucose-lowering therapies, which may limit generalizability to advanced, treated disease states. The exclusion was necessary to avoid confounding by medication effects on glucose and insulin levels, but it means that our findings are most applicable to treatment-naïve individuals with normal-to-moderately elevated glucose levels. The inclusion of treatment-naïve participants with newly identified diabetes-range fasting glucose (13% of the cohort) enhances the generalizability to the early detection setting, where screening for IR could identify individuals at risk before formal T2D diagnosis.

Fourth, testosterone was not measured, limiting our ability to directly adjust for andropausal effects. As discussed, testosterone plays a critical role in glucose metabolism and IR in men, and its absence from the feature set may have reduced model performance. The effects of testosterone are likely captured indirectly through age and BMI, but direct measurement would provide more precise estimates and could reveal sex-specific pathways.

Fifth, the validity of HOMA-IR varies in older populations with impaired glucose tolerance [30]. HOMA-IR was originally developed and validated in relatively healthy populations, and its accuracy may be reduced in individuals with impaired beta-cell function or advanced age. This may partially explain the lower performance in older participants and highlights the need for alternative IR measures in geriatric populations.

Finally, the apparent scale difference in SHAP values reflects feature measurement units rather than purely predictive importance; thus, we relied on cross-metric triangulation (SHAP + permutation importance + Elastic Net coefficients) for robust feature ranking. The large SHAP values for BMI (1.326) compared to VOCs (0.207 for methanol) partly reflect the larger scale of BMI measurements (range 15–45) compared to VOC concentrations (typically 1–100 ppb). Normalization of SHAP values by feature standard deviation or use of permutation importance helped mitigate this issue and confirmed that the clinical markers, particularly BMI and TG, are indeed the dominant predictors.

## 5. Conclusions

This study demonstrates that machine learning approaches can achieve acceptable predictive performance for insulin resistance in men. While traditional markers like BMI, TG, and HDL-C were the primary drivers of the models, the integration of specific VOCs (e.g., methanol, formic acid) provided additional, orthogonal insights into metabolic dysregulation. VOC profiling holds potential as a non-invasive, complementary early screening tool for male populations. Future longitudinal studies incorporating direct hormonal measurements and external validation are required to establish the temporal utility of breath analysis in clinical risk stratification.

## Figures and Tables

**Table 1:** Hyperparameter tuning grid and final optimized parameters for the XGBoost model.

Hyperparameter	Search_Grid_Values	Final_Optimized_Value
max_depth	3, 5, 6, 7	6
eta (learning_rate)	0.01, 0.05, 0.1	0.05
subsample	0.8	0.8
colsample_bytree	0.8	0.8
min_child_weight	1, 3, 5	3
gamma	0	0
nrounds	100, 200, 300, 389	389

Note: Hyperparameters of the XGBoost model were optimized using a predefined grid search strategy. The table summarizes the candidate values evaluated for each hyperparameter and the final values selected for model development. The optimized model used a maximum tree depth of 6, learning rate (eta) of 0.05, sub-sample ratio of 0.8, column sampling ratio of 0.8, minimum child weight of 3, gamma value of 0, and 389 boosting iterations (nrounds). These parameters were selected based on their ability to minimize prediction error on the training data while maintaining generalizability.

**Table 2:** The demographic, biochemistry, and lifestyle features of the participants.

Variables	Unit	Mean ± SD
**Continuous Variables**		
Age	years	45.59 ± 12.71
Body Mass Index	kg/m^2^	24.62 ± 3.85
Systolic Blood Pressure	mmHg	123.22 ± 15.82
Diastolic Blood Pressure	mmHg	80.82 ± 10.62
Glutamate Pyruvate Transaminase	U/L	33.99 ± 31.71
Estimated Glomerular Filtration Rate	mL/min/1.73 m^2^	82.42 ± 14.10
Uric Acid	mg/dL	6.52 ± 1.28
Triglycerides	mg/dL	121.68 ± 88.83
High-Density Lipoprotein Cholesterol	mg/dL	49.59 ± 12.27
Low-Density Lipoprotein Cholesterol	mg/dL	127.39 ± 36.60
Drinking status	-	5.55 ± 15.97
Smoking status	-	5.02 ± 13.64
Exercise habits	-	7.68 ± 9.32
Fasting glucose	mg/dL	98.4 ± 18.2
HbA1c	%	5.6 ± 0.7
**Categorical Variables**		
Marital status	Single	334 (29.7%)
	Married	790 (70.3%)
Education level	Illiterate	1 (0.1%)
	Elementary school	11 (1.0%)
	Junior high school	33 (3.0%)
	High school	130 (11.6%)
	Junior college	179 (16.1%)
	University	484 (43.4%)
	Graduate school or above	277 (24.8%)
Sleep time	Less than 4 h	11 (0.9%)
	4–6 h	307 (26.6%)
	6–7 h	550 (47.7%)
	7–8 h	251 (21.8%)
	8–9 h	28 (2.4%)
	More than 9 h	7 (0.6%)
Glucose metabolism status	Normal fasting glucose (<100 mg/dL)	742/1258 (59.0%)
Prediabetes (fasting glucose 100–125 mg/dL)		352/1258 (28.0%)
Newly identified diabetes-range fasting glucose (≥126 mg/dL): 164/1258 (13.0%)		164/1258 (13.0%)
**Dependent Variable**		
HOMA-IR	-	2.11 ± 2.35

Note: There were 134 missing data for marital status (10.7%). Cumulative lifestyle indices were calculated as follows: drinking area = alcohol proof (%) × amount × duration (years); smoking area = cigarettes/day × duration (years); exercise area = intensity × hours/week × duration (years).

**Table 3:** Performance comparison of machine learning models for HOMA-IR prediction.

Model	Set	R^2^	RMSE	MAE
Elastic Net	Training	0.244435	5.557619	2.986466
Elastic Net	Test	0.188579	6.187272	3.197647
MARS	Training	0.551338	4.282646	2.600624
MARS	Test	0.236668	6.00113	3.319457
Random Forest	Training	0.880169	2.213287	1.152163
Random Forest	Test	0.32272	5.652757	2.94201
XGBoost	Training	0.655371	3.753431	2.277268
XGBoost	Test	0.293758	5.772355	2.984033

Note: R^2^: coefficient of determination; RMSE: root mean square error; MAE: mean absolute error; MARS: multivariate adaptive regression splines; XGBoost: eXtreme Gradient Boosting. All models were trained on 70% of the data and tested on the remaining 30%. Training set metrics represent performance on the finalized, full training set configuration post-hyperparameter optimization; internal 5-fold cross-validation tuning metrics are reported separately in Section 3.2 text.

**Table 4:** Top 20 features ranked by mean |SHAP| value from XGBoost model.

Rank	Feature	Mean abs SHAP
1	BMI	1.345
2	TG	0.844
3	HDL-C	0.553
4	GPT	0.492
5	eGFR	0.332
6	SBP	0.223
7	Methanol	0.202
8	UA	0.187
9	1-Butyne	0.184
10	Age	0.180
11	DBP	0.176
12	Acetone	0.172
13	Ethanedial	0.146
14	Formic acid	0.121
15	1-Propanol	0.115
16	Heptane	0.112
17	Cyclohexane	0.099
18	Butyl acetate	0.093
19	Methyl acetate	0.091
20	Styrene	0.088

Note: SHAP: SHapley Additive exPlanations. Features are ranked by their mean absolute SHAP values, indicating the average impact on model output magnitude. Higher values indicate greater importance in predicting HOMA-IR. BMI: body mass index; TG: triglycerides; HDL-C: high-density lipoprotein cholesterol; GPT: glutamate pyruvate transaminase; eGFR: estimated glomerular filtration rate; SBP: systolic blood pressure; UA: uric acid; DBP: diastolic blood pressure.

**Table 5:** Elastic Net coefficients for features with non-zero contributions.

Feature	Coefficient	Absolute Coefficient
BMI	216.30	216.30
TG	68.34	68.34
HDL-C	−59.84	59.84
GPT	28.74	28.74
Butanone	−15.80	15.80
o-Xylene	12.55	12.55
Ethanedial	−8.38	8.38
Cyclohexane	−4.35	4.35
Limonene	4.09	4.09
(E)-2-Nonenal	−1.41	1.41

Note: BMI: body mass index; TG: triglycerides; HDL-C: high-density lipoprotein cholesterol; GPT: glutamate pyruvate transaminase. Positive coefficients indicate positive association with HOMA-IR, while negative coefficients indicate inverse association. Only features with non-zero coefficients after elastic net regularization are shown.

**Table 6:** Incremental predictive value of VOCs over conventional clinical–biochemical markers.

Model Type	Features Included	Test Set R^2^	Test Set RMSE	Test Set MAE	ΔR^2^ (vs. Conventional)	*p*-Value *
Conventional-only	Age, BMI, BP, lipids, GPT, eGFR, UA, HbA1c, lifestyle	0.265	5.81	3.01	Reference	-
VOC-only	All selected VOC features	0.112	6.54	3.45	−0.153	<0.001
Combined model	Conventional + VOCs	0.294	5.77	2.98	0.029	0.012

Note: Performance comparison of three XGBoost models evaluated on the independent test set to formally assess the incremental predictive value of volatile organic compounds (VOCs). The conventional-only model includes standard clinical and biochemical markers (age, BMI, blood pressure, lipids, GPT, eGFR, UA, HbA1c, and lifestyle variables). Abbreviations: BMI: body mass index; BP: blood pressure; GPT: glutamate pyruvate transaminase; eGFR: estimated glomerular filtration rate; UA: uric acid; HbA1c: glycated hemoglobin. * *p*-value from the bootstrap permutation test comparing each model with the conventional-only reference model.

**Table 7:** Diagnostic performance of machine learning models for binary classification of insulin resistance using a HOMA-IR cutoff of 2.5.

Model	AUC	Sensitivity	Specificity	Accuracy	PPV	NPV	F1-Score
Elastic Net	0.869	0.857	0.881	0.869	0.878	0.860	0.867
MARS	0.860	0.848	0.872	0.860	0.869	0.852	0.858
Random Forest	0.969	0.957	0.981	0.969	0.981	0.958	0.969
XGBoost	0.988	1.000	0.942	0.960	0.885	1.000	0.939

Note: Performances of Elastic Net, MARS, Random Forest, and XGBoost models for identifying insulin resistance, defined as HOMA-IR ≥ 2.5. Model discrimination was evaluated using the area under the receiver operating characteristic curve (AUC), sensitivity, specificity, accuracy, positive predictive value (PPV), negative predictive value (NPV), and F1-score in the independent test dataset (*n* = 377). Abbreviations: MARS: Multivariate Adaptive Regression Splines; XGBoost: eXtreme Gradient Boosting. AUC: area under the curve; PPV: positive predictive value; NPV: negative predictive value; IR: insulin resistance. Higher values indicate better discriminative ability. Values for XGBoost were derived from the confusion matrix in Supplementary Table S1.

**Table 8:** Comparison of binary classification performance between conventional-only and combined models for insulin resistance prediction.

Model	AUC	Sensitivity	Specificity	Accuracy	PPV	NPV	F1 Score	*p* Value
Conventional-only	0.965	0.957	0.923	0.94	0.862	0.987	0.912	Reference
Combined Model	0.988	1	0.942	0.971	0.885	1	0.939	0.008

Note: Performance comparison between the conventional clinical model (including age, BMI, blood pressure, lipids, GPT, eGFR, UA, HbA1c, and lifestyle variables) and the combined model (conventional markers + VOCs) for binary classification of insulin resistance (HOMA-IR ≥ 2.5). AUC: area under the curve; PPV: positive predictive value; NPV: negative predictive value.

## Data Availability

Data available on request due to privacy/ethical restrictions.

## Supplementary Materials

The following supporting information can be downloaded at: https://www.mdpi.com/article/10.3390/biomedicines14081751/s1, Table S1. Raw counts of true negatives (TN), false positives (FP), false negatives (FN), and true positives (TP) for the combined model predicting insulin resistance (HOMA-IR ≥ 2.5) in the 30% held-out test set (*n* = 377). IR: Insulin resistance. The model achieved PPV = 88.5% (116/131) and NPV = 100% (246/246). Figure S1. Calibration plot. Note: Calibration plot demonstrating the agreement between predicted probabilities and observed probabilities for the combined XGBoost model. The dashed diagonal line represents perfect calibration. The red LOESS smoothing curve indicates strong agreement between predicted risk and observed outcomes in the binary classification of insulin resistance. Figure S2. Decision curve analysis: combined vs. conventional-only Model.
