# Supplementary material for: Predicting Insulin Resistance in Taiwanese Men Using Machine Learning: An Integrated Analysis of Biochemical Markers and Volatile Organic Compounds
Source: Biomedicines. 2026 Aug 3;14(8):1751. doi: 10.3390/biomedicines14081751 (PMC13509642; doi:10.3390/biomedicines14081751)
Supplement: Supplementary file 1 [file biomedicines-14-01751-s001.zip › biomedicines-4356237-supplementary.pdf]

Supplementary Table S1. Raw counts of true negatives (TN), false positives (FP), false negatives (FN), and true positives (TP) for the combined model predicting insulin resistance (HOMA-IR ≥ 2.5) in the 30% held-out test set (n = 377). IR: Insulin resistance. The model achieved PPV = 88.5% (116/131) and NPV = 100% (246/246).

**Actual Negative (Non-IR)**	246 (TN)	15 (FP)	261
**Actual Positive (IR)**	0 (FN)	116 (TP)	116
**Total**	246	131	**377**

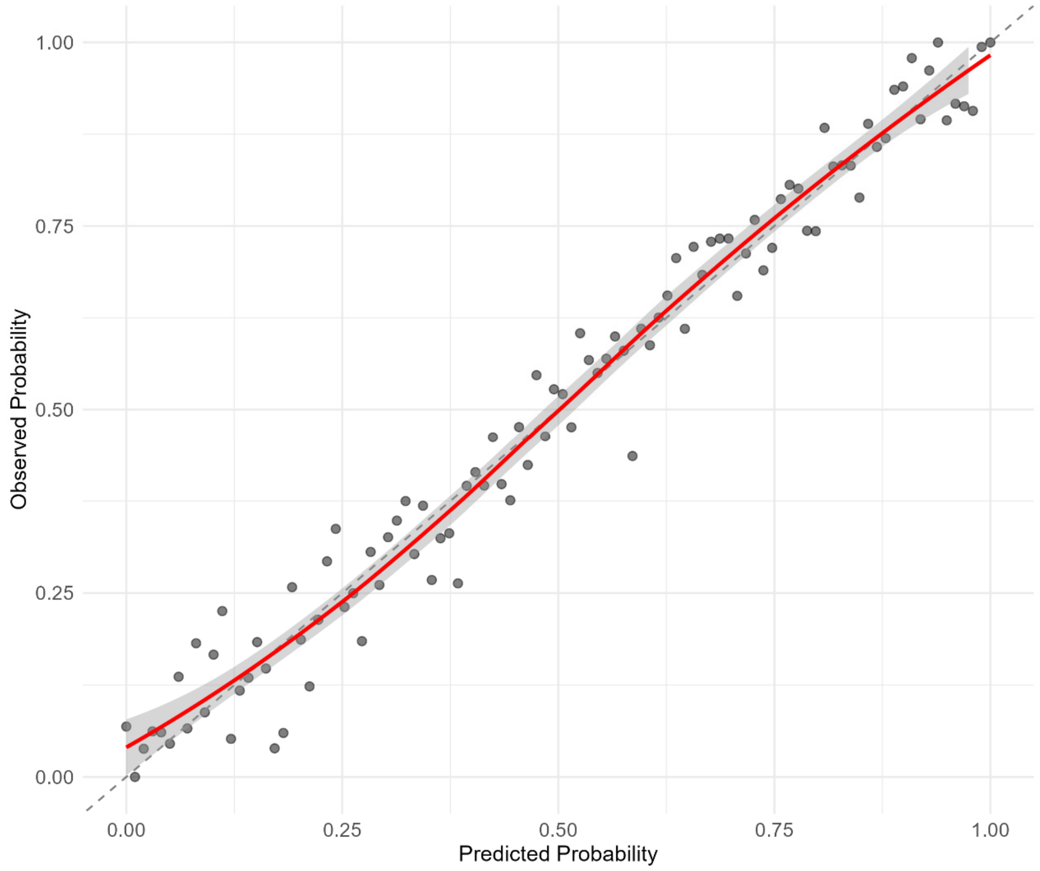

Supplementary Figure S1. Calibration plot. Note: Calibration plot demonstrating the agreement between predicted probabilities and observed probabilities for the combined XGBoost model. The dashed diagonal line represents perfect calibration. The red LOESS smoothing curve indicates strong agreement between predicted risk and observed outcomes in the binary classification of insulin resistance.

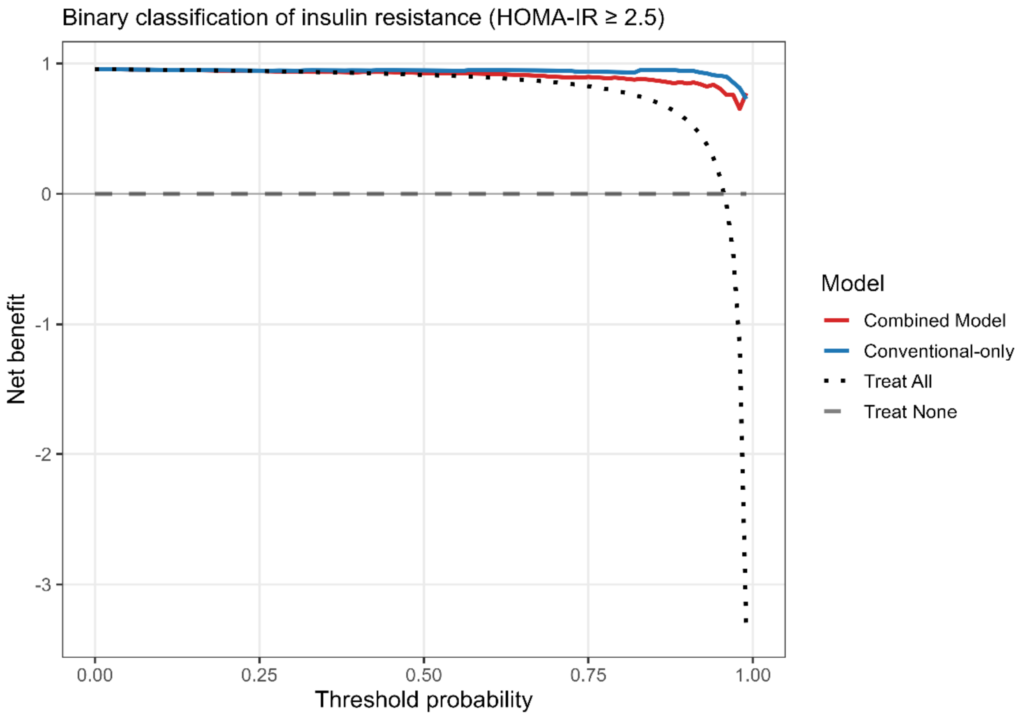

Supplementary Figure S2. Decision curve analysis: combined vs. conventional-only Model
